# Efficient genetic editing of human intestinal organoids using ribonucleoprotein-based CRISPR

**DOI:** 10.1242/dmm.050279

**Published:** 2023-10-05

**Authors:** Nefeli Skoufou-Papoutsaki, Sam Adler, Paula D'Santos, Liz Mannion, Shenay Mehmed, Richard Kemp, Amy Smith, Francesca Perrone, Komal Nayak, Alasdair Russell, Matthias Zilbauer, Douglas J. Winton

**Affiliations:** ^1^ CRUK Cambridge Institute, Cambridge CB2 0RE, UK; ^2^ Wellcome-MRC Cambridge Stem Cell Institute, Cambridge CB2 0RE, UK

**Keywords:** *PTEN*, Genome editing, Organoids

## Abstract

Organoids, combined with genetic editing strategies, have the potential to offer rapid and efficient investigation of gene function in many models of human disease. However, to date, the editing efficiency of organoids with the use of non-viral electroporation methods has only been up to 30%, with implications for the subsequent need for selection, including turnaround time and exhaustion or adaptation of the organoid population. Here, we describe an efficient method for intestinal organoid editing using a ribonucleoprotein-based CRISPR approach. Editing efficiencies of up to 98% in target genes were robustly achieved across different gut anatomical locations and developmental timepoints from multiple patient samples with no observed off-target editing. The method allowed us to study the effect of loss of the tumour suppressor gene *PTEN* in normal human intestinal cells. Analysis of *PTEN*-deficient organoids defined phenotypes that likely relate to its tumour suppressive function *in vivo*, such as a proliferative advantage and increased organoid budding. Transcriptional profiling revealed differential expression of genes in pathways commonly known to be associated with *PTEN* loss, including mTORC1 activation.

## Teaser

**Summary:** High-efficiency ribonucleoprotein-mediated genome editing enables rapid functional studies in human organoids by avoiding the need for clonal selection.

## INTRODUCTION

Epithelial cells isolated from their resident tissue can be instructed to form three-dimensional (3D) self-organising and renewing organoid structures when placed in 3D matrices and provided with appropriate niche signals to maintain proliferative stem cells (Sato et al., 2009). Organoids have bridged the gap between *in vivo* tissue-based observations in human and mouse and those made in cell lines cultured in two dimensions. They have diverse applications, from mechanistic studies of tissue development to disease modelling and regenerative medicine (Rossi et al., 2018).

Organoid research has matured partly due to the concurrent development of technologies for genetic engineering, enabling studies of gene function (Fujii et al., 2015; Fessler et al., 2016; Kraiczy et al., 2017; van Rijn et al., 2018). Edited organoids have also been used for xenotransplantation approaches (Drost et al., 2015; Matano et al., 2015; Fumagalli et al., 2017). There are two main methods for introducing genome editing components into the cells: viral (retrovirus, lentivirus) and non-viral (electroporation and lipofection) (Teriyapirom et al., 2021). Viral methods could traditionally reach efficiencies of 30-50%, but more recent modifications of the transduction method have achieved efficiencies of 80-100% (Pirona et al., 2020; Gu et al., 2022). However, viral methods have associated biosafety and permanent integration issues, whereas non-viral methods are simpler, minimising the time required for the production of the viral particles, which can take up to 2-3 months (Lin et al., 2022). For the non-viral methods, electroporation has been found to result in higher organoid-editing efficiencies (Fujii et al., 2015).

**Fig. 1.**
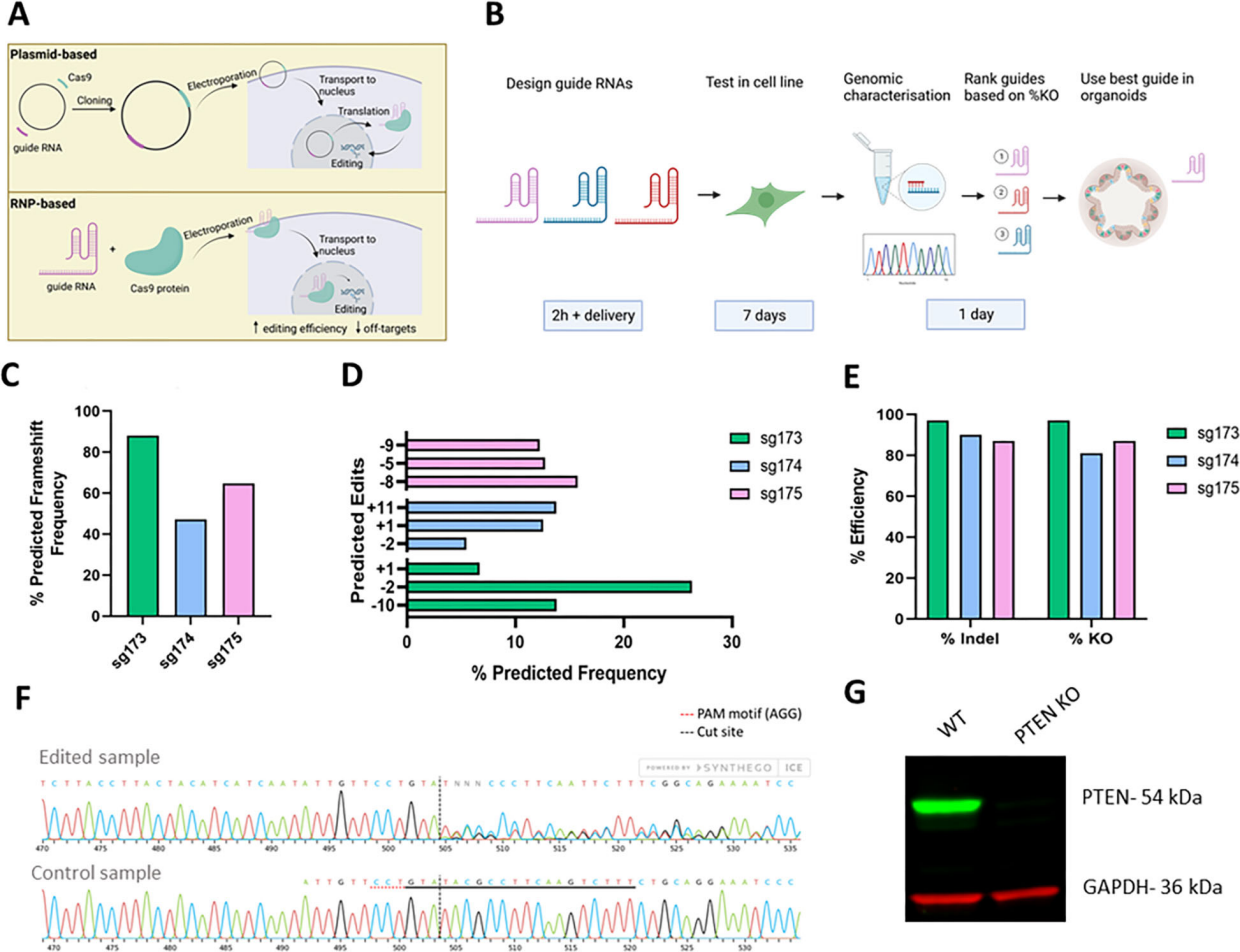
**Experimental approach and optimisation of guide RNAs in cells.** (A) Plasmid and ribonucleoprotein (RNP)-based approach for CRISPR-Cas9 genome editing. (B) Experimental approach for selection of the best-performing guide RNA in organoids and timeline. (C) Expected frameshift frequency of three *PTEN* guide RNAs. (D) Top three predicted edits for three *PTEN* guide RNAs. (E) Testing of three *PTEN* guide RNAs in MDA-MB-231 cells. ‘% Indel’ includes all edits and ‘% KO’ indicates edits that will lead to a frameshift. (F) Sanger trace for edited sample and control. The black dotted line indicates the cut site and the red dotted line indicates the protospacer adjacent motif. (G) Western blot for PTEN in wild-type (WT) and *PTEN* knockout (KO) cells.

There are two approaches for genetic editing reagents based around delivery of encoding vectors or ribonucleoprotein (RNP) (Fig. 1A). For the RNP-based approach, a synthetic guide RNA is complexed with a purified recombinant Cas9 protein. This approach eliminates the need to translate the Cas9 protein and has reduced off-target effects (Kim et al., 2014). More specifically, a 2.5- to 28-fold reduction in off-target effects has been reported with an RNP method compared to plasmid-based methods (Liang et al., 2015; Zhang et al., 2021). For these reasons, RNP-based approaches are being rapidly adopted by the genome editing community (DeWitt et al., 2017). However, so far, vector-based approaches have been mainly used to edit human organoids, resulting in efficiencies of only 10-30% (Fujii et al., 2015). This requires subsequent selection of the edited population, using antibiotics or growth factors, and increases the time required for the generation of engineered populations, risking their exhaustion or adaptation during expansion.

Here, we describe an efficient method for the generation of genetic knockout (KO) human intestinal organoids using an RNP-based CRISPR approach with the use of electroporation to deliver the editing components. Owing to the high editing efficiencies, no subsequent clone selection is required. The method was applied to study the effect of the loss of the tumour suppressor gene *PTEN*. Molecular and phenotypic characterisation of the edited organoids functionally validated the approach and allowed behaviours likely relating to the tumour suppressive role of *PTEN* to be described.

## RESULTS

### Generation and validation of *PTEN* KO organoids

For RNP-mediated editing, a custom-made synthetic guide RNA is complexed with a recombinant Cas9 protein (Fig. 1A). Guide RNAs were designed using online tools Benchling and Indelphi with a predicted on-target score >40 (Doench et al., 2016), off-target score >80 (Hsu et al., 2013) and frameshifting score >80 (Shen et al., 2018). Three guides were designed per target gene to disrupt an early exon. Guides were first tested in a cell line to identify those with the best editing efficiency (% KO) as inferred following the deconvolution of Sanger sequencing using Inference of CRISPR Edits (ICE) software (Conant et al., 2022) (Fig. 1B).

*PTEN* was selected as an initial target gene for disruption to generate a KO organoid model. It is an important tumour suppressor gene mutated in 6% of colorectal cancers (CRCs), and germline variants in *PTEN* are causative of PTEN hamartoma tumour syndrome (Hobert and Eng, 2009; Cerami et al., 2012). The *PTEN* coding region is 8515 bp, and the gene is located on chromosome 10q23.3 (Cunningham et al., 2022). The guide RNAs were designed to target exon 2 of *PTEN*. Of note, *PTEN* contains a highly conserved pseudogene, *PTENP1*, located at chromosome 9p21, with 98% sequence homology with the coding region of functional *PTEN* (Dahia et al., 1998). Based on the online design tool, *PTENP1* was also predicted to be targeted with the designed guide RNAs owing to this high sequence homology. When tested in cells, high editing efficiencies of up to 97% were seen with one of the *PTEN* guides (sg173) (Fig. 1C-F). Loss of PTEN protein expression was confirmed in the edited cells (Fig. 1G).

**Fig. 2.**
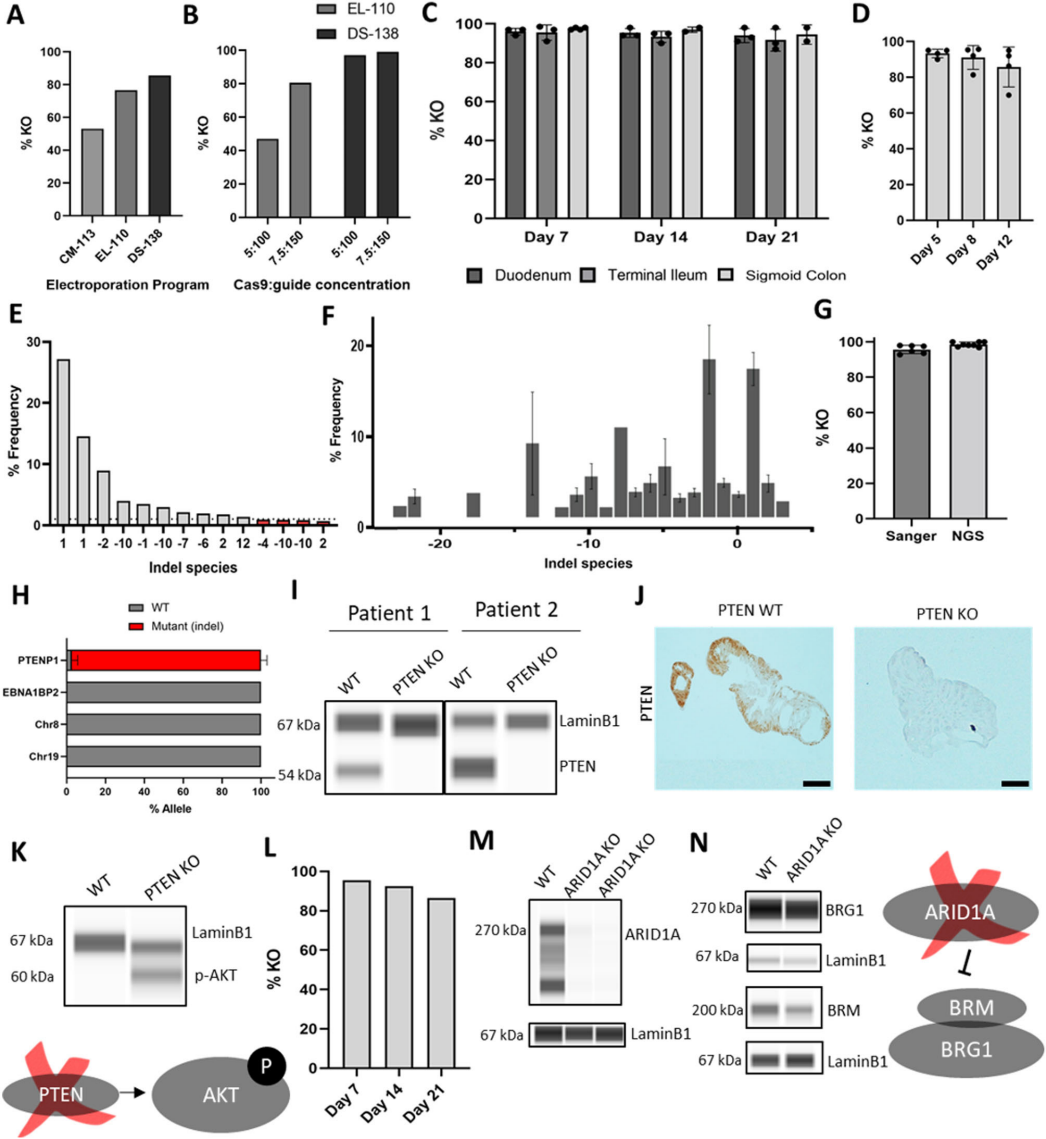
**Efficient editing of human intestinal organoids.** (A) Testing of different Lonza electroporation programmes using 50,000 cells. (B) The two most efficient programmes, EL-110 and DS-138, tested on 100,000 cells with 7.5 μg and 150 pmol or 5 μg and 100 pmol Cas9:guide RNA concentration. (C) PTEN electroporation, using 100,000 cells, DS-138 programme and lower Cas9 concentration, on three gut segments from three different paediatric patients. Percentage KO monitored over three passages (*n*=3). (D) Foetal organoid PTEN edited using the same conditions as in C (*n*=4). (E) Example of allelic species for one sample used for next-generation sequencing (NGS). The dotted line indicates 1% allelic frequency, which was the set cutoff. Red bars indicate allelic species below the cutoff used. No WT reads were called. (F) Percentage frequency of different allelic species within all the *PTEN* KO samples submitted for NGS. Data were from three patients and three timepoints, and include species with >1% allele frequency. (G) Percentage KO assessed using NGS and Sanger sequencing (*n*=6). (H) Off-target effects of *PTEN* guide RNA assessed using NGS. Percentage WT (same size as predicted product) or mutant allele (contains insertions or deletions leading to a different-sized product). Data are from three patients. Off-target genomic coordinates for *PTENP1*: chr8:140155102, chr19:55504388. (I) Output of Wes (Bio-Techne) for PTEN protein in WT and *PTEN* KO organoids (*n*=2). (J) Immunohistochemistry for PTEN in WT and *PTEN* KO organoid formalin-fixed paraffin-embedded sections (*n*=3). Scale bar: 400 μm. (K) Output of Wes (Bio-Techne) for p-AKT (Ser473) in WT and *PTEN* KO organoids (*n*=3). (L) Organoid electroporation using *ARID1A* guide RNA. Percentage KO monitored over three passages. Data are from three technical replicates from one patient (*n*=3). (M) Output of Wes (Bio-Techne) for ARID1A in WT and *ARID1A* KO organoids. Data are from two technical replicates (*n*=2). (N) Output of Wes (Bio-Techne) for BRG1 and BRM in WT and *ARID1A* KO organoids (*n*=3). Graphs show mean±s.d.

The best-performing guide identified in cells was tested in human intestinal organoids. Different electroporation conditions, such as the commercially provided electroporation programmes (see Materials and Methods), number of cells, and varying Cas9 and guide RNA concentrations comprising the targeting complex, were tested to identify the combination leading to the highest editing efficiency (Fig. 2A,B). The optimal condition was the use of 100,000 cells with the DS-138 programme using Lonza P3 buffer. Similar editing efficiencies of ∼95% KO were detected with different complex concentrations for this programme, and the lower concentration, 5 μg:100 pmol, was used subsequently. Efficiencies of up to 98% were seen in three different patients and gut segments – duodenum, terminal ileum and sigmoid colon – and remained stable over 3 weeks in culture (Fig. 2C). High editing efficiencies were also observed in colonic organoids of a different developmental stage, that is, human foetal organoids (Fig. 2D).

For validation of the genetic KO, samples were submitted for next-generation sequencing (NGS) covering the area targeted by the guide RNA. For the NGS analysis, all allelic species with a frequency higher than 1% were considered (Fig. 2E). Species with indels leading to a different product size than the wild-type (WT) allele were considered mutant and were used to calculate the KO score. Even with this more sensitive sequencing method, the frequency of WT alleles across the edited samples was only up to 3% (Fig. 2F). There was good correlation between the KO score estimated from the Sanger sequencing and NGS (Fig. 2G). In addition, primers were designed to cover the top regions predicted to have potential off-target binding of the guide RNA based on the guide design tool (Benchling). These were composed of the top three off-target areas, including the *PTEN* pseudogene, *PTENP1*, which has 100% sequence similarity with the *PTEN* guide RNA, and two intergenic regions, in chromosomes 8 and 19. In addition, the highest off-target area within a gene, *EBNA1BP2*, was included. The anticipated editing of the *PTEN* pseudogene was observed, but none of the other areas were targeted by the guide RNA, with all the reads being of the WT sequence (Fig. 2H). This shows that the method is specifically targeting the areas in the genome with the exact guide RNA sequence.

Next, the effect of PTEN loss at the protein level was assessed. Loss of PTEN protein expression was observed using Wes^TM^ (Bio-Techne) automated capillary-based immunodetection and by immunohistochemistry (Fig. 2I,J). A downstream target of PTEN, phosphorylated (p)-AKT, previously described as increased upon *PTEN* loss (Takao et al., 2018), was confirmed to be elevated in *PTEN* KO organoids (Fig. 2K).

### Independent validation of organoid editing by targeting *ARID1A*

Electroporation conditions were next applied with a different guide, targeting another tumour suppressor gene, *ARID1A*. High levels of KO efficiency were also observed (Fig. 2L). Loss of ARID1A protein expression was confirmed (Fig. 2M). ARID1A acts to regulate ATPases (BRM and BRG1) of the large ATP-dependent SWI/SNF chromatin-remodelling complexes involved in transcriptional regulation of gene expression and that are present in mutually exclusive complexes. To establish whether there were downstream consequences of loss of ARID1A, the expression of both ATPases was investigated. Of note, protein levels of BRM, but not BRG1, were reduced, demonstrating a functional consequence of *ARID1A* loss (Fig. 2N).

**Fig. 3.**
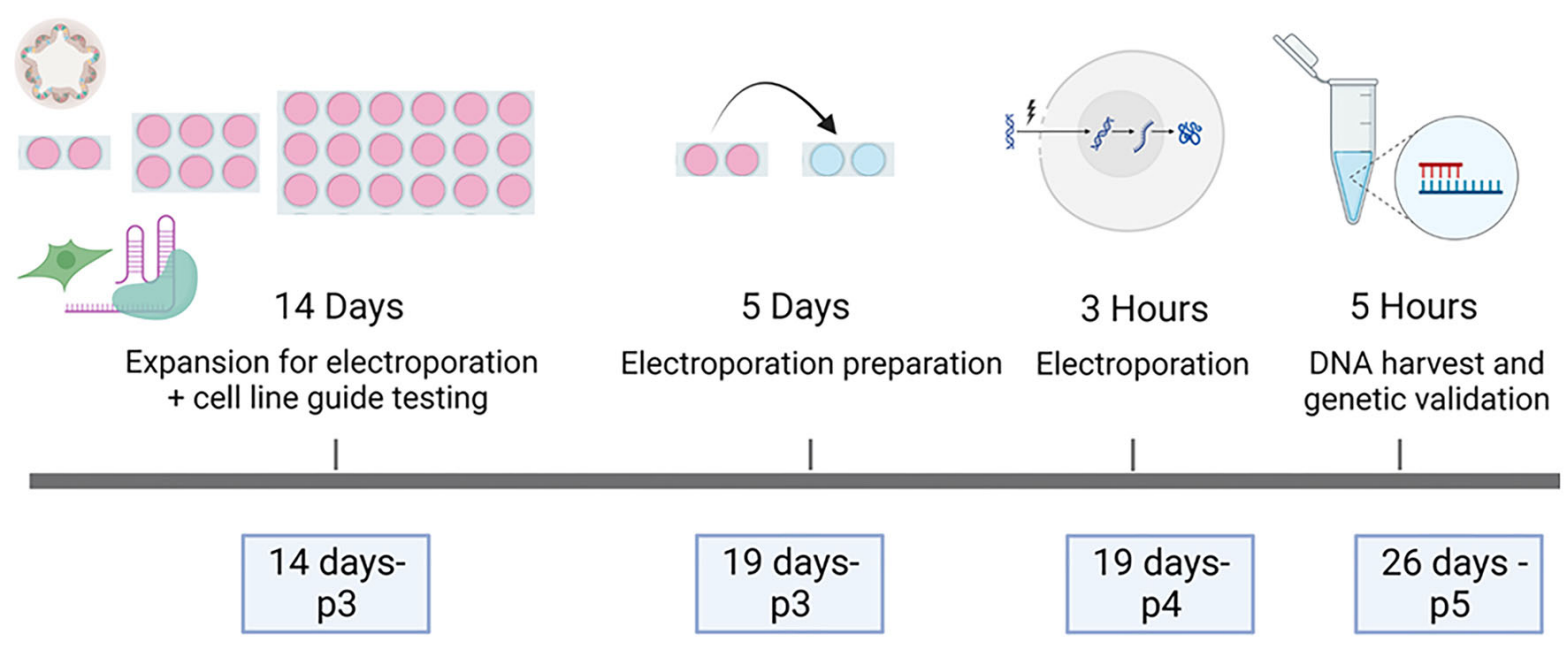
**Outline of organoid electroporation process and timeline.** KO organoids can be generated within 26 days and five passages (p), starting from two wells of intestinal biopsies. This timing includes also the testing of target guide RNAs in cell lines.

These findings confirmed high KO efficiency with different gut segments, organoids from different developmental timings and different guide RNAs. With the method described here, edited organoids can be generated within 26 days, including the time required for adequate expansion of the culture and the design and testing of the synthetic guides (Fig. 3).

### *PTEN* KO organoids exhibit increased budding and proliferative advantage

The phenotypic effect of *PTEN* loss in a human sigmoid colon organoid model was next assessed to explore how the normal intestinal cells might be affected in a way that could relate to cancer development, which could act as functional validation of the model.

**Fig. 4.**
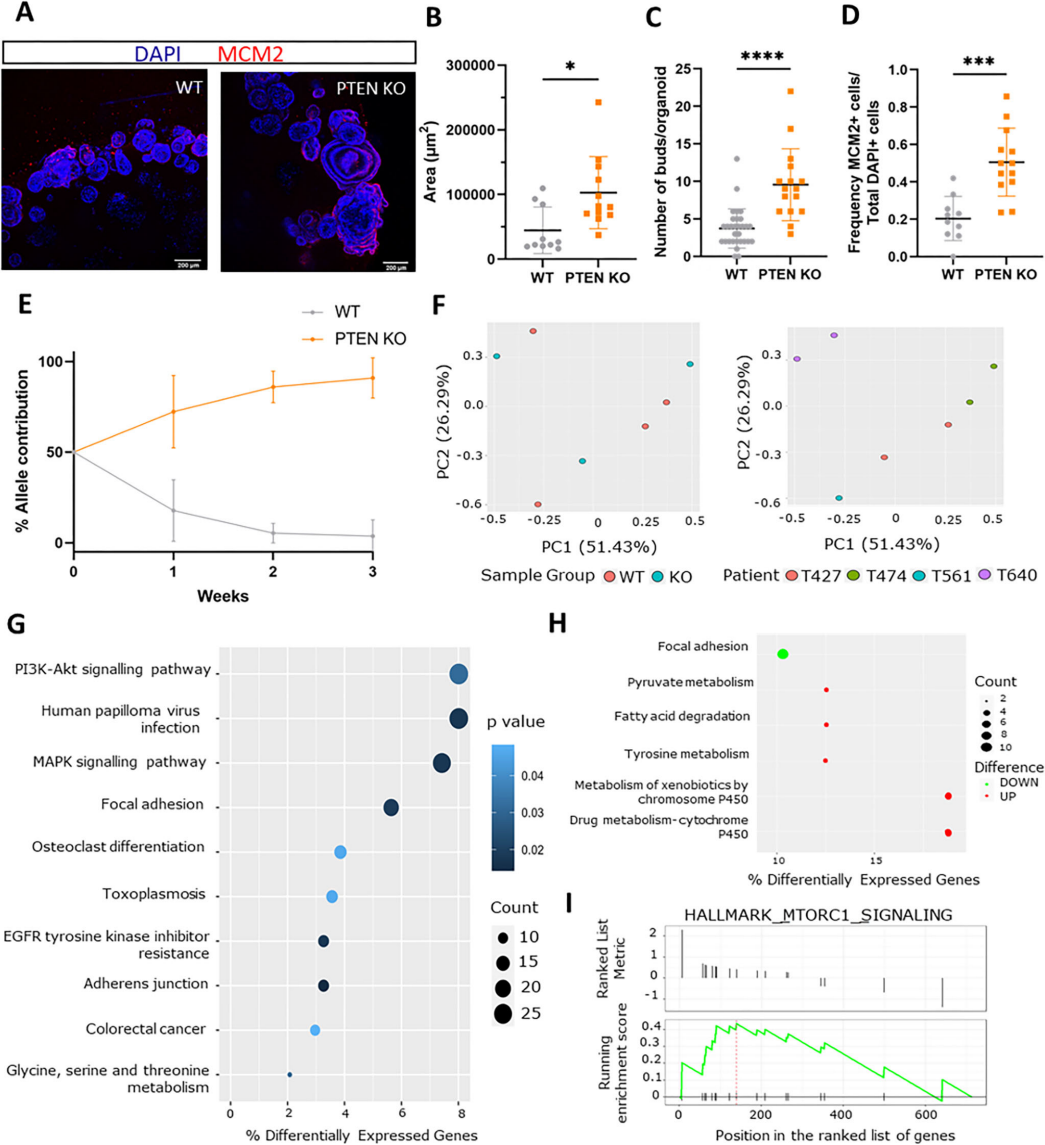
**Phenotypic and molecular characterisation of *PTEN* KO organoids functionally validates genetic KO.** (A) Wholemount staining of organoids. Shown as maximum projection. (B) Organoid area (μm^2^). Mann–Whitney test. *P=0.0112; *n*=2. (C) Number of buds per whole organoid. Mann–Whitney test. ****P<0.0001; *n*=2. In B and C, data are from two patients and three technical replicates. (D) Frequency of MCM2-positive cells. Data are from three patients. Unpaired two-tailed Student's *t*-test. ***P=0.0002; *n*=3. (E) Competition assay. WT and *PTEN* KO allele contribution over 3 weeks (21 days). Data are from three patients (*n*=3). (F) Principal component (PC) analysis based on patient ID and genotype. (G) Differentially expressed Kyoto Encyclopedia of Genes and Genomes (KEGG) pathways. (H) Most highly differentially expressed KEGG pathways (log fold change >0.7 and <−0.7). (I) Gene set enrichment analysis Hallmark pathways: mTORC1 pathway (enrichment score, 0.44; *P*=0.047). Graphs show mean±s.d.

*PTEN* KO organoids appeared morphologically larger, with higher cell number, and exhibited an increased number of budding structures compared to WT organoids (Fig. 4A-C; Fig. S1A-D). An increase in cell proliferation was also observed upon PTEN loss, as indicated by a higher proportion of cycling cells marked by the S-phase marker MCM2 (Fig. 4D; Fig. S1E).

A competition assay was set up to assess whether *PTEN* KO cells have a competitive advantage over WT cells in organoid cultures (Fig. 4E; Fig. S1F). Indeed, after mixing *PTEN* KO cells with WT cells in a 50:50 ratio, the KO cells completely took over within 3 weeks in culture, suggesting a direct proliferative advantage or possibly that they act to inhibit the growth of WT organoids. To investigate the latter, WT cells were grown in *PTEN* KO conditioned medium derived isogenically (Fig. S1G-I). No effect on the WT organoid numbers or their size was observed, suggesting that *PTEN* KO cells outcompete WT cells in a cell-autonomous way, with a likely contribution from increased proliferation.

### Transcriptomic characterisation of *PTEN* KO organoids functionally validates genetic editing

Next, bulk RNA sequencing (RNA-seq) was performed to correlate the molecular changes with phenotypic alterations in *PTEN* KO sigmoid colon organoids and validate the editing on the transcriptomic level. Three isogenic pairs of WT and *PTEN* KO patient-derived organoids were profiled. Principal component analysis clustered organoids based on the originating patient rather than the genotype, suggesting a strong patient effect, which was incorporated in the differential expression model (Fig. 4F). There were, in total, 730 differentially expressed genes, with 290 of them upregulated and 440 downregulated (false discovery rate <0.05).

Pathway analysis of differentially expressed genes indicated enrichment for cancer-associated Kyoto Encyclopedia of Genes and Genomes (KEGG) pathways such as the PI3K-AKT pathway, of which PTEN is a negative regulator, in addition to the MAPK signalling pathway and CRC (Fig. 4G). When focusing on the genes with the highest fold changes, focal adhesion was the top downregulated pathway in *PTEN* KO organoids (log FC <−0.7), while alternative metabolic pathways, such as fatty acid degradation and tyrosine metabolism, were included in the most highly upregulated pathways (log FC >0.7) (Fig. 4H; Fig. S2A).

There was a general enrichment of the regulation of cell proliferation processes, with some important genes, such as *TGFB1* and *GCNT2*, being differentially expressed. Orthogonal validation of some proliferation and metabolism targets was performed using quantitative PCR (qPCR) and Wes (Bio-Techne) (Fig. S2B-D).

In addition, the mTORC1 pathway was found to be enriched in the *PTEN* KO organoids (Fig. 4I). This pathway is an important regulator of cell proliferation and metabolism (Hay and Sonenberg, 2004; Guertin and Sabatini, 2007). mTORC1 is also known to be important for controlling cell size, with activated mTORC1 leading to larger cells (Fingar et al., 2002). The size of a cell is known to be proportionate to the size of its nucleus (Edens et al., 2013), and, indeed, *PTEN* KO nuclei were found to be larger than WT nuclei (Fig. S2E), in line with the predicted effect of mTORC1 activation.

Finally, in terms of processes that might relate to the increased budding phenotype, enrichment of the mammary branching during breast development was implicated in the *PTEN* KO organoids, with three of six genes (*AREG*, *PHB2* and *TGFB1*) known to be involved in the process being differentially expressed (Fig. S2F).

## DISCUSSION

Primary organoid cultures contain much of the diversity present in the donor. Avoiding the need for selection following gene editing serves to minimise the time required for expansion and retains more of this biological diversity. The efficiency of the genome editing method described here was similar to viral delivery methods, without the associated biohazard and mutagenesis issues. The use of the RNP method allowed an almost 3-fold increase in the editing efficiency compared to previously published electroporation-based delivery methods using a plasmid-based approach (Fujii et al., 2015). The RNP-based CRISPR method was shown to be extremely robust, working with similar efficiencies for organoids taken from gut segments of different anatomical locations and developmental timepoints from multiple patients and for different target genes. Gene edits remained constant over time in culture, and the editing was restricted to regions with 100% sequence homology with the guide RNA. Although not tested, the method will likely translate to different epithelial organoid systems. A recently published protocol for the RNP-based CRISPR approach, applied to mutate the tumour suppressor gene *FBXW7* in intestinal organoids, also employed an RNP-based method, with a combination of three synthetic guide RNAs (Chan et al., 2023). The current study, where a single guide RNA was sufficient for comparable levels of genome editing, complements this report and additionally showcases the application of the resultant organoid models by thorough molecular and functional characterisation. The generation of an *ARID1A* genetic KO demonstrated a reduction in the levels of one of the two downstream active mediators, BRM but not BRG1, which has not, to our knowledge, been previously identified. Of note, similar editing efficiencies as the ones described here were recently reported using a novel technique of peptide-assisted genome editing (Zhang et al., 2023). This was described as a rapid and robust way to edit primary cells, so it would be interesting to investigate its applicability to the organoid field.

The morphological and transcriptomic characterisation of the *PTEN* KO organoids validated the editing event, with previously reported changes in signalling pathways and processes associated with loss of *PTEN* also seen in the organoid model. Loss of *PTEN* was associated with a proliferative advantage, metabolic rewiring and reduction in the process of focal adhesion.

To gain insight into the process of cancer development, previous studies have attempted to replicate and test the order of gene mutation in CRC using organoids by sequential editing of the relevant events (Matano et al., 2015). This required repeated growth-factor-based selection of the edited organoids, which allowed the analysis of, for example, SMAD4 mutations that circumvent a specific organoid growth dependency, but not PTEN. The method employed here avoids the need for clonal selection, thus changing the calculus; now any tumour suppressor gene can be mutated to determine its functional role at different stages of the neoplastic process. Illustratively, the involvement of PTEN in the focal adhesion pathway via direct regulation of focal adhesion kinase (FAK; PTK2) has been reported (Tamura et al., 1998, 1999). These studies described the downregulation of focal adhesion formations in NIH3T3 cell lines engineered to overexpress *PTEN*, in apparent contradiction to the effect shown here, where the same effect resulted from *PTEN* loss. This difference demonstrates the need to test gene, and cancer driver, functions in biologically relevant contexts, such as those provided by primary organoids that can be efficiently gene edited.

## MATERIALS AND METHODS

The in-detail description of the organoid electroporation using RNP-based CRISPR method protocol can be found at protocols.io (https://dx.doi.org/10.17504/protocols.io.5qpvor74xv4o/v1).

### Human intestinal samples

Intestinal mucosal biopsies were collected from sigmoid colon, terminal ileum and duodenum from children under 16 years old undergoing diagnostic colonoscopy at Addenbrooke's Hospital, Cambridge, UK. This study was conducted with informed patient and/or carer consent as appropriate, and with full ethical approval (REC-12/EE/0265). Foetal intestine was obtained with informed consent, and with ethical approval (REC-96/085), from elective terminations at 8-12 weeks’ gestational age. All clinical investigations were conducted in accordance to the principles expressed in the Declaration of Helsinki.

### Human intestinal organoid culture and maintenance

Intestinal organoids were cultured in ADF+++ medium, referred to here as WENRAFI (Table S1). Medium was replaced every 2-3 days. The standard organoid culture conditions were adapted from those previously described by replacing the p38 inhibitor with IGF1 and FGF2 (Fujii et al., 2018).

On passaging, organoids were disaggregated by intense pipetting. Following centrifugation (500 ***g***, 4 min for all steps), the pellet was reseeded in fresh Matrigel (20 μl per well; Corning, 356231) in a 48-well plate with 250 μl WENRAFI plus 10 μM of the ROCK inhibitor Y-27632 (xxxxxsupplier), a ROCK inhibitor, for the first 24 h.

### Design of synthetic guide RNAs and primers

Guide RNAs were ordered from Synthego (Table S2). Three guide RNAs were designed per target using Benchling and Indelphi (Shen et al., 2018). Guides with predicted high on-target (>40), off-target (>80) and frameshift (>80%) scores were selected, preferentially targeting an early exon of the gene. Their KO efficiency was first assessed in cells. The guide yielding the highest KO per gene was taken forward for use in organoids.

Primers were designed on Primer 3 so that the target region is 150 bp upstream and downstream from the expected edit site to allow successful comparison of Sanger sequencing traces, with products ranging from 500 bp to 800 bp (Table S2).

### Electroporation-related organoid culture

Organoids were passaged 5 days prior to electroporation. The medium was changed to reduced ENAFI medium (without WNT and RSPO) supplemented with 5 μM CHIR99021 (Bio-Techne, 4423) and 10 μM Y-27632 (ENAFI-CY+; Bio-Techne, 1254) 48 h before electroporation and then changed to ENAFI-CY+ containing 1.25% (vol/vol) dimethyl sulfoxide (DMSO) 24 h before electroporation (Table S3). For electroporation, organoids were seeded in ENAFI-CY+ medium with 1.25% (vol/vol) DMSO. The next day, medium was changed to WENRAFI+10 μM Y-27632.

### Electroporation using RNP

Parts of the protocol have been modified from Fujii et al. (2015) for RNP delivery in cells using electroporation.

On electroporation, the culture medium was removed, and 500 μl TrypLE Express Enzyme (Thermo Fisher Scientific, 12605010) supplemented with 10 μM Y-27632 was added to each well. Matrigel was scraped, and the organoids were transferred into Falcon tubes and placed in a 37°C water bath for 30 min, with vigorous pipetting every 5 min to achieve single-cell dissociation. ADF basal medium Invitrogen, 12634-028 was added up to 10 ml, and the tubes were centrifuged. True Cut Cas9 v2 protein (Thermo Fisher Scientific, A36499; 500 μg at 5 μg/μl) and full-length, synthetic guide RNAs (previously reconstituted with water to 100 pmol/μl) were thawed on ice. The supernatant was aspirated, and the cells were re-suspended in 0.5 ml Opti-MEM medium (Thermo Fisher Scientific, 31985062). The number of cells was counted (i.e. 100,000 cells/condition were used, unless stated otherwise). The RNP complex was made by mixing 1 μl of 5 μg Cas9 and 1 μl of 100 pmol guide (1:3.3 ratio). The complex was incubated for 20 min at room temperature. A negative, no-Cas9, control and at least three technical replicates were always included. Cells were centrifuged, the supernatant was aspirated, and 300 μl PBS was added. Cells were centrifuged, the supernatant was aspirated, and the pellet was resuspended in the appropriate volume of P3 supplement buffer (20 μl per condition by mixing 16.4 μl P3 buffer and 3.6 μl supplement 1; Lonza, V4XP-3032). The cells were transferred into 16-well nucleovette strips (Lonza). After 10 min at room temperature, electroporation was performed on an Amaxa 4D Nucleofector (Lonza) with the DS-138 custom programme unless otherwise stated. The cells were then incubated for 10 min at 37°C and then 80 μl pre-warmed medium was added to the electroporation chambers. Cells were centrifuged, supernatant was discarded, and the pellet was resuspended in 20 μl Matrigel per condition and seeded in 48-well plates (100,000 cells per well).

### Screening of gene-edited organoids

One-third of organoids in each well were harvested for screening at 7 days post-electroporation. DNA was extracted using an Arcturus DNA PicoPure kit (Thermo Fisher Scientific, KIT0103). Organoids were centrifuged, and the pellet was resuspended in 20 μl Proteinase K buffer from the Arcturus DNA PicoPure kit. They were incubated for 3 h at 65°C, followed by 10 min incubation at 95°C.

Genomic DNA was used for PCR amplification of the targeted exon in the gene of interest using Q5 polymerase enzyme (NEB, M0491) according to the manufacturer's instructions, with 2 μl organoid lysate in a 50 μl reaction. The product was cleaned using a PCR cleanup kit (Zymo Research, D4013) and submitted for Sanger sequencing in both directions.

To quantify a gene KO score, the Sanger sequencing traces were deconvoluted using the Inference of CRISPR Edits (ICE) tool by Synthego (Conant et al., 2022).

### Targeted-amplicon NGS

DNA from three patients and two technical replicates, over three timepoints, was subjected to targeted amplicon NGS. Regions of interest included the area targeted by the *PTEN* guide RNA and the top off-target regions as predicted by Benchling. Primers were ordered with the addition of the CS1 and CS2 Illumina adapters. To amplify the regions of interest, PCR was performed using Phusion polymerase (NEB). Unique Fluidigm sample barcodes were added using a Fast Start High Fidelity PCR System (Roche). The library was balanced based on the Bioanalyser molarity. A Column-based Clean & Concentrator Kit (Zymo Research) was used for PCR cleanup. Primer dimers were eliminated by broad range (200-400 bp) size selection using a Blue Pippin (Sage Science), and samples were submitted for 150 bp paired-end Miseq Nano Illumina sequencing.

For the analysis, a paired-end assembler for Illumina sequences, PANDAseq, was used to merge corresponding forward and reverse reads into an *in silico* amplicon (Masella et al., 2012). The top ten different species that started and ended with the correct primer sequence were identified, their frequency was calculated, and their size was noted. Based on the predicted amplicon size, they were then classified as mutant reads with indels or WT reads with no difference in the predicted product length.

### Organoid formalin-fixed paraffin-embedded plug generation

Organoids were fixed in 4% paraformaldehyde for 20 min at 4°C. Giemsa dye was added (1:10 in 70% ethanol) for 1 h. After washing with 70% ethanol, organoids were resuspended in 2% pre-heated low-melting-point agarose and transferred on the cap of an Eppendorf, which was used as a mould. The plug was removed and 10% neutral buffered formalin was added and left overnight at 4°C. Plugs were embedded in paraffin and cut into 5 μm sections.

### Immunodetection

Antigen retrieval was performed using citrate buffer (10 mM sodium citrate, pH 6) with a laboratory pressure cooker (125°C for 2.5 min and then 91°C for 10 s). Blocking was performed with 10% donkey or goat serum (Dako) or 3% bovine serum albumin (Sigma, A9647), incubating for 30 min. Primary antibody was incubated overnight at 4°C (Table S4). The next day, secondary antibody [JacksonImmunoResearch biotin-SP-conjugated AffiniPure donkey anti-mouse or anti-rabbit (AB_2340785 and AB_2340593, respectively), 1:500 dilution for immunohistochemistry; Alexa fluorophore-conjugated antibody, 1:200 for immunofluorescence] was added for 40 min [with 1 μg/ml 4′,6-diamidino-2-phenylindole (DAPI; Thermo Fisher Scientific, D1306) if doing immunofluorescence]. For immunohistochemistry, pre-made Vector ABC mix (Vectastain Elite ABC Reagent, Vector Laboratories) was added for 40 min. Slides were developed using DAB and DAB-substrate chromogen system (Dako).

### Organoid wholemount staining

Organoids were single-cell dissociated using TrypLE Express Enzyme (Thermo Fisher Scientific, 12605010), and 15,000 cells were seeded per well in eight-well chambers (ibidi, 80826) suitable for confocal imaging. Wholemount staining was performed 7 days post single-cell dissociation using a standard immunofluorescence wholemount protocol.

### Image analysis

For the wholemount images, ImageJ was used to quantify the number of DAPI-positive cells per organoid. The area of interest was circled, and the threshold was adjusted. The numbers of particles were then analysed automatically. The organoid area was also quantified. Three *z*-stacks per image were analysed, and the average was calculated.

### Protein analysis using organoid protein lysates

Organoids were spun down and lysed in RIPA buffer (Thermo Fisher Scientific, 89900) with the addition of Halt Phosphatase Inhibitor Cocktail (Thermo Fisher Scientific, 78420). The Wes^TM^ (Bio-Techne) automated separation module was used according to the manufacturer's instructions. Antibodies used are shown in  Table S4.

### Organoid competition assay

Organoids were single-cell dissociated using TrypLE, and a total of 15,000 cells were seeded per well. One-third of organoids in a well were harvested to extract DNA 7 days post single-cell dissociation and then 14 and 21 days after. Synthego ICE was used to deconvolute the mixed trace and infer the WT and KO percentages. After the day 7 timepoint, organoids were passaged normally without single-cell dissociation.

### Conditioned medium experiment

Organoids were single-cell dissociated using TrypLE, and a total of 10,000 cells were seeded per well. WT cells were cultured either with PTEN KO or WT conditioned medium with the addition of growth factors (EGF, NOG, FGF1, IGF2, WNT, RSPO). Images were taken every 3 h over 7 days in an Incucyte S3 Sartorius.

### RNA extraction

Frozen WT and *PTEN* KO organoids were thawed at the same time and expanded for 1 week. They were single-cell dissociated, and, 7 days later, RNA was extracted using an Arcturus PicoPure RNA extraction kit (Thermo Fisher Scientific, KIT0204) according to the manufacturer's instructions.

### Bulk RNA-seq library preparation

Library preparation was performed using a Stranded mRNA Prep kit (Illumina, 20040532) according to the manufacturer's instructions. Samples were submitted for sequencing in the Illumina Novaseq platform with 50 bp paired-end reads. One of the WT samples (T561) consistently failed the library preparation and was excluded.

### RNA-seq data analysis

Differential expression analysis was performed using DESeq2. An interaction model was used to identify differentially expressed genes.

Downstream analyses were then performed in R using standard RNA-seq packages – clusterProfiler, pathview and msigdbr – for gene set enrichment analysis (GSEA) (Subramanian et al., 2005; Yu et al., 2012; Luo and Brouwer, 2013). KEGG pathway analysis focused on the most highly differentially expressed genes (fold change >0.7 or <−0.7). GSEA was performed using Hallmark pathways. Enrichment in Gene Ontology (GO) Biological Processes analysis was performed using the GO online resource, based on the top 500 differentially expressed genes in the dataset (based on descending *P*-value).

### qPCR validation

cDNA conversion was performed using ProtoScript II RT standard protocol (NEB, M0368) and Random Primer Mix (NEB, S1330S). qPCR was performed using TaqMan Fast Universal PCR Master Mix (Thermo Fisher Scientific, 4352042), and Taqman probes and primers for *G0S2* (Thermo Fisher Scientific, Hs00274783_s1), *ADH1C* (Thermo Fisher Scientific, Hs02383872_s1), *NUPR1* (Thermo Fisher Scientific, Hs01044304_g1) and *GCNT2* (Thermo Fisher Scientific, Hs00377334_m1), according to the manufacturer's instructions.

### Statistical analysis

Statistical analysis was performed in GraphPad Prism. Unpaired Student's two tailed *t*-test was performed if the data followed a normal distribution; otherwise, Mann-Whitney *U* test was performed.

## Supplementary Material

10.1242/dmm.050279_sup1 Supplementary information
