# Supplementary material for: Efficient genetic editing of human intestinal organoids using ribonucleoprotein-based CRISPR
Source: Dis Model Mech. 2023 Oct 5;16(10):dmm050279. doi: 10.1242/dmm.050279 (PMC10565108; doi:10.1242/dmm.050279)
Supplement: Supplementary information [file dmm-16-050279-s1.pdf]

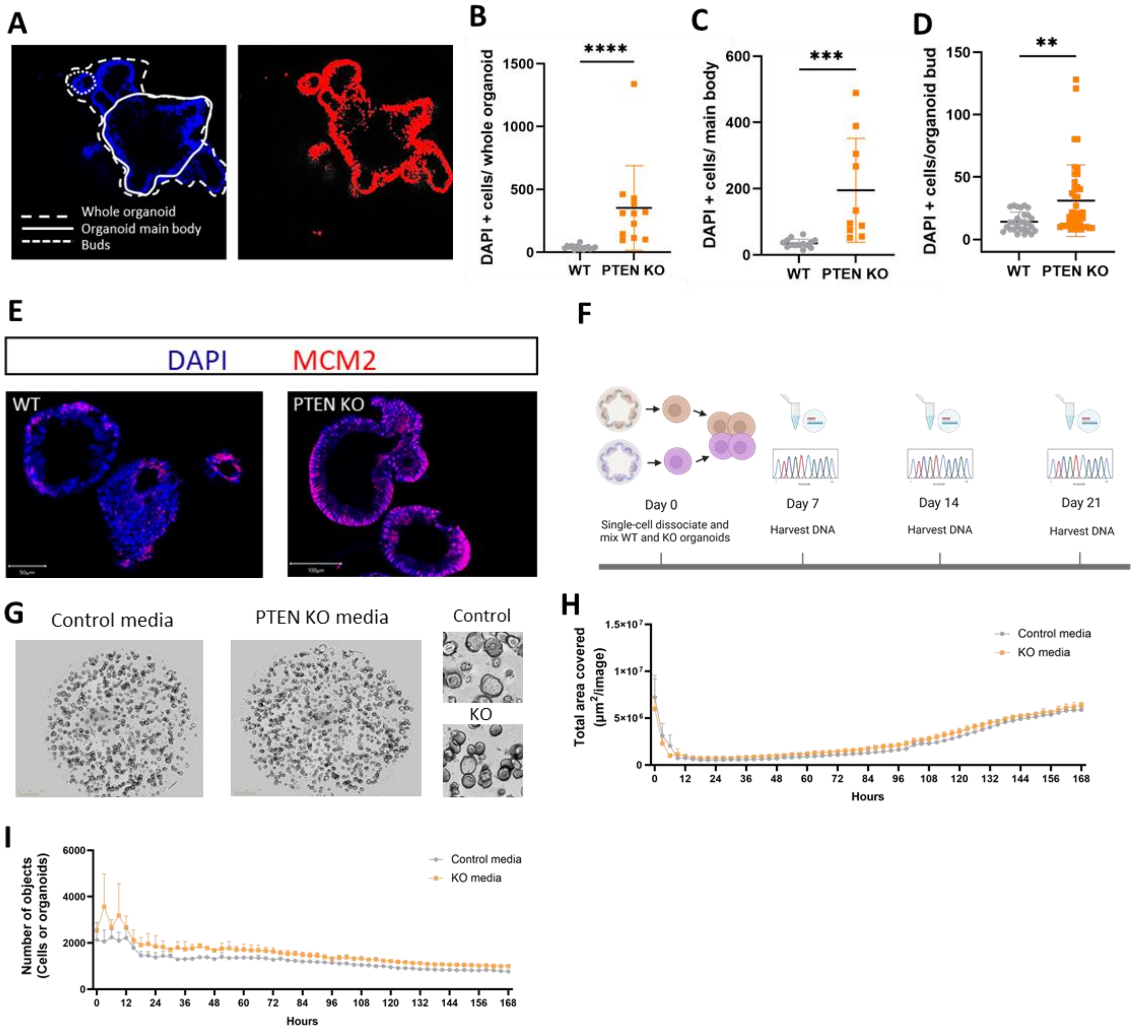

Fig. S1. Phenotypic characterisation of PTEN KO organoids. (A) Definition of organoid regions and counting of cells in Fiji. (B) Number of DAPI positive cells per whole organoid. Mann-Whitney test. p-value: < 0.0001. (C) Number of DAPI positive cells per main body. Unpaired t-test. p= 0.0009. (D) Number of DAPI positive cells per organoid bud. Mann-Whitney test. p-value= 0.0019. (E) Immunofluorescence MCM2 staining on FFPE organoid sections. (F) Schematic of experimental outline for competition assay. (G) Images of WT organoids cultured with WT or PTEN KO conditioned media, 7 days post single-cell dissociation. Inlet showing top right corner of each well in higher magnification. (H) Quantification of organoid numbers in conditioned media experiment. (I) Quantification of organoid area in conditioned media experiment. N= data from three technical replicates.

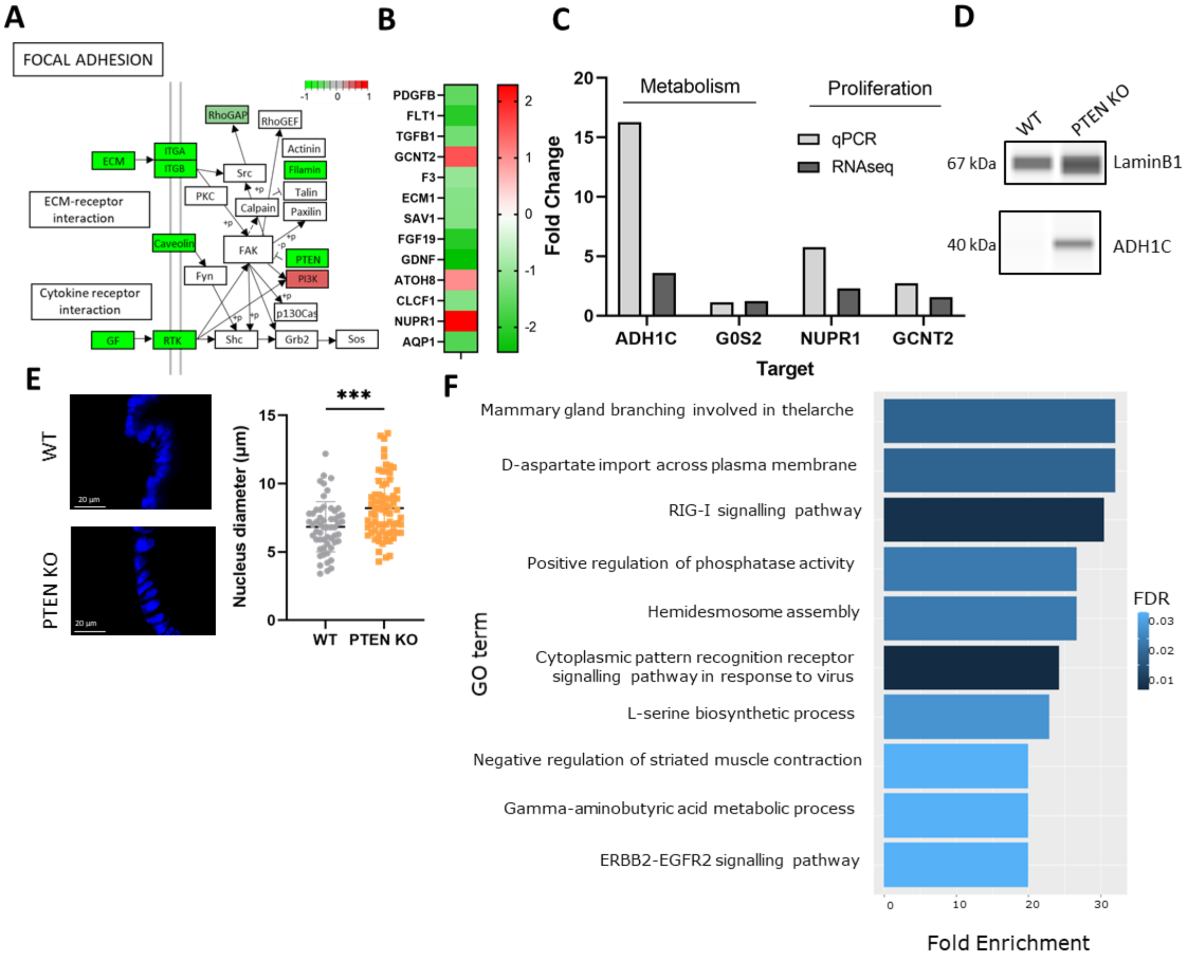

Fig. S2. Transcriptomic profiling of PTEN KO organoids. (A) Focal Adhesion pathway with highlighted genes being differentially expressed. Focus on the membrane side. (B) Regulation of cell proliferation identified as one of the gene ontology processes that is enriched in the dataset. Showing genes involved in regulation of cell proliferation with biggest fold change (>1 and <-1). (C) qPCR validation of fatty acid degradation targets ADH1C and G0S2 and proliferation regulators NUPR1 and GCNT2. Comparison with RNAseq fold change. Data from three patients. (D) Wes (Biotechne) protein validation of fatty acid target ADH1C. (E) Quantification of cell size. Nuclei diameter (µm). (F) Gene Set Enrichment Analysis of Gene Ontology terms based on top 500 differentially expressed genes ranked by ascending p-value. Showing top 10 Gene Ontology (GO) terms.

Table S1. WENRAFI media composition. Modified from Fujii et al., 2018. Normal organoid maintenance medium.

Optimised organoid media (replacement of p38i)-WENRAFI	Stock concentration	Volume	Final concentration
**ADF+++**	pure	13.08	
**Wnt3a conditioned medium**	pure	25 ml	50%
**R-spo conditioned medium**	pure	10 ml	20%
**Primocin (Invivogen #ant-pm-1)**	50mg/ml (500x)	100µl	500 µg/mL
**B-27® Supplement (Thermofisher #17504-044)**	50x	1000 µl	1x
**Nicotinamide (Sigma #N0636, in water)**	1 M (100x)	500 µl	10 mM
**N-Acetylcysteine (Sigma # A9165, in water)**	500 mM (400x)	125 µl	1.25 mM
**A3801 (Tocris #2939, in DMSO)**	5 mM (10,000x)	5 µl	500 nM
**mEGF (Thermofisher Biosource #PMG8043)**	100 ng/µl (2,000x)	25 µl	50 ng/mL
**mNoggin (Peprotech #250-38)**	100 ng/µl (1,000x)	50 µl	100 ng/mL
**IGF-1 (Biolegend, 590904)**	100 ng/µl	50 µl	100 ng/mL
**FGF-2 (Peprotech, #100-18B)**	100 ng/µl	25 µl	50 ng/mL
Total		50 mL	

Table S2. Guide RNA sequence for guides used in organoids and primer sequence used for screening.

**Target**	**Guide sequence**	**F primer sequence**	**R primer sequence**
PTEN	AAAGACTTGAAGGCGTATAC	GGCAGGTGTCAATTTTGGGG	CCTTGGTACACCCAGCGAT
ARID1A	CGGTACCCGATGACCATGCA	GCCTTTGTTTATACCCGGCC	CCACTGCCTTTCATCCCATC

Table S3. ENAFI media composition. Used for organoid electroporation.

ENAFI (EGF, Noggin, ADF, FGF2, IGF1)	Stock concentration	Final concentration	ENAFI+ Y+Chir (48h before)	ENAFI+ Y+Chir+DMSO (24h before and elec day)
**ADF+++**	Pure		24010	23697.5
**Primocin (Invivogen, #ant-pm-1)**	50mg/ml (500x)	500 µg/mL	50	50
**B-27® Supplement (Thermofisher, #17504-044)**	50x	1x	500	500
**Nicotinamide (Sigma #N0636, in water)**	1 M (100x)	10 mM	250	250
**N-Acetylcysteine (Sigma # A9165, in water)**	500 mM (400x)	1.25 mM	62.5	62.5
**A3801 (Tocris #2939, in DMSO)**	5 mM (10,000x)	500 nM	2.5	2.5
**mEGF (Thermofisher Biosource, #PMG8043)**	100 ng/µl (2,000x)	50 ng/mL	12.5	12.5
**mNoggin (Peprotech, #250-38)**	100 ng/µl (1,000x)	100 ng/mL	25	25
**IGF-1 (Biolegend, 590904)**	100 ng/µl	100 ng/mL	25	25
**FGF-2 (Peprotech, #100-18B)**	100 ng/µl	50 ng/mL	12.5	12.5
**Y-27632 (STEM Cell Technologies, 73302)**	10 mM	10 uM	25	25
**CHIR99021 (Ref)**	10 mM	5 uM	25	25
**DMSO**		1.25%		312.5
Total			25mL	25mL

Table S4. Primary antibodies used for immunohistochemistry (IHC), immunofluorescence (IF) or Wes™.

Protein	Supplier	Reference	Titre	Application
**PTEN**	Cell Signaling	#9552	1:300	IHC
**MCM2**	BioRad	MCA1859	1:300	IF
**Lamin B1**	Cell Signaling	#12586S	1:2000	Wes
**p-Akt**	Cell Signaling	#4060S	1:50	Wes
**GAPDH**	Cell Signaling	#5174S	1:50	Wes
**PTEN**	Cell Signaling	#9552	1:100	Wes
